# Design and implementation of load intensity monitoring platform supported by big data technology in stage training for women’s sitting volleyball

**DOI:** 10.1038/s41598-023-50057-9

**Published:** 2023-12-16

**Authors:** Zhijun Liang, Chen Liang

**Affiliations:** China volleyball college, Beijing sport university, Beijing, 100084 China

**Keywords:** Computational science, Computer science, Information technology, Software, Statistics, Health care, Data processing

## Abstract

This study aims to discuss the load intensity monitoring in the training process of sitting volleyball, to help coaches understand the training status of athletes, and to provide a scientific basis for the follow-up training plan. Through big data technology, the physiological changes of athletes can be more accurately grasped. This includes classification and summary of exercise load intensity and experimental study of the relationship between heart rate and rating perceived exertion (RPE). Through monitoring the training process of a provincial women’s sitting volleyball team, it is found that there is a significant positive correlation between athletes’ RPE and average heart rate. This result shows that by monitoring the change in heart rate and RPE of athletes, athletes' training state and physical condition can be more accurately understood. The results reveal that through the use of big data technology and monitoring experiments, it is found that heart rate and RPE are effective monitoring indicators, which can scientifically reflect the load intensity during sitting volleyball training. The conclusions provide coaches with a more scientific basis for making training plans and useful references for sports involving people with disabilities.

## Introduction

### Research background

Exercise load is one of the most important aspects of contemporary sports training, which plays a crucial role in the ability and performance of athletes in competition. With the continuous improvement of the competition level, the intensity of the training load is also increasing, which puts forward higher requirements on the physical fitness, athletic ability, and psychological quality of athletes^1^. However, because of its unique characteristics, sitting volleyball is not widely known. At present, the age of sitting volleyball players is increasing, which has an impact on the quality and function of their bodies. Monitoring the load intensity of the sitting volleyball team in training is vital to ensure the training effect of the players. Nowadays, due to the rapid development of information technology, especially the emergence of big data technology, it is possible to track athletes' physical conditions in real time^2^. By continuously monitoring an athlete's heart rate using a heart rate monitor, coaches can develop training schedules and plans that best suit the athlete's unique physical condition. Nevertheless, because sitting volleyball does not require a lot of running and jumping, the accuracy of heart rate monitoring is not high. Therefore, the Rating Perceived Exertion (RPE) scale based on heart rate monitoring can more accurately monitor the training load intensity of sitting volleyball players^3^.

### Literature review

There was relatively little research on the disability sport of sitting volleyball in China and other countries. Gaweł and Zwierzchowska^4^ performed objective and subjective measurements in 34 Polish sitting volleyball players to determine the effect of compensation mechanisms on the prevalence of trunk lordosis and musculoskeletal pain, and to assess the interrelationship between these components. Their research showed a moderate association among lumbar discomfort, thoracic lordosis, and lumbar posterior fovea^4^. Krzysztofik et al.^5^ studied the effect of the percentage of predetermined speed loss on subsequent bench press throwing performance of 12 disabled sitting volleyball players when lifting their legs or feet off the ground for bench press. Bench press was found to improve bench press throw performance with leg lift^5^. Ahmadi et al.^6^ investigated the correlation between isokinetic force and isometric grip strength of the scapular rotator muscle. By using Biodex isokinetic instrument to measure shoulder joint rotation at 180/s and 0/s in 50 sitting volleyball players, a correlation was found between isokinetic grip strength and isokinetic peak torque and total work in seated volleyball players^6^. To check the validity of the current classification method, Cavedon et al.^7^ analyzed linear anthropometry, physical health status, and sprint performance of sitting volleyball players and studied them in three groups: disabled athletes, minimally disabled athletes, and healthy athletes^7^. Wiliński et al.^8^ conducted grip strength experiments on nine players and used the Spearman correlation coefficient to evaluate the results to explore the relationship between the strength level of sitting volleyball players and their sports efficiency. They found that the strength of sitting volleyball players was related to their athletic performance, and when the grip was strong, the upper limb was more likely to move when off the ground^8^. To measure the reaction time of sitting volleyball players, Yazdani and Pakzad Hassanluo^9^ created and manufactured a system that can evaluate reaction time in six different directions^9^. These studies contributed to the development of sitting volleyball by examining the lumbar spine, strength and reaction time of players. However, research was still limited in terms of training load intensity. Moreover, sitting volleyball didn’t take off in China until relatively recently, and few studies tracked the severity of the burden athletes were put under during exercise. Consequently, this study created a monitoring platform that used monitoring indicators to track the intensity of sitting volleyball training to improve the whole sport.

On the basis of the background information provided above, this study analyzes the terms used to describe exercise load and load intensity as well as the classification of exercise load. It also discusses the monitoring indicators of load intensity and selects heart rate and RPE as the monitoring indicators. The research object is a provincial female queue, and training load intensity is observed. The study of the women's sitting volleyball team’s exercise load intensity can offer suggestions for choosing sports training monitoring indicators for impaired athletes.

## Research methodology

### Concept definition of exercise load intensity

**Figure 1:**
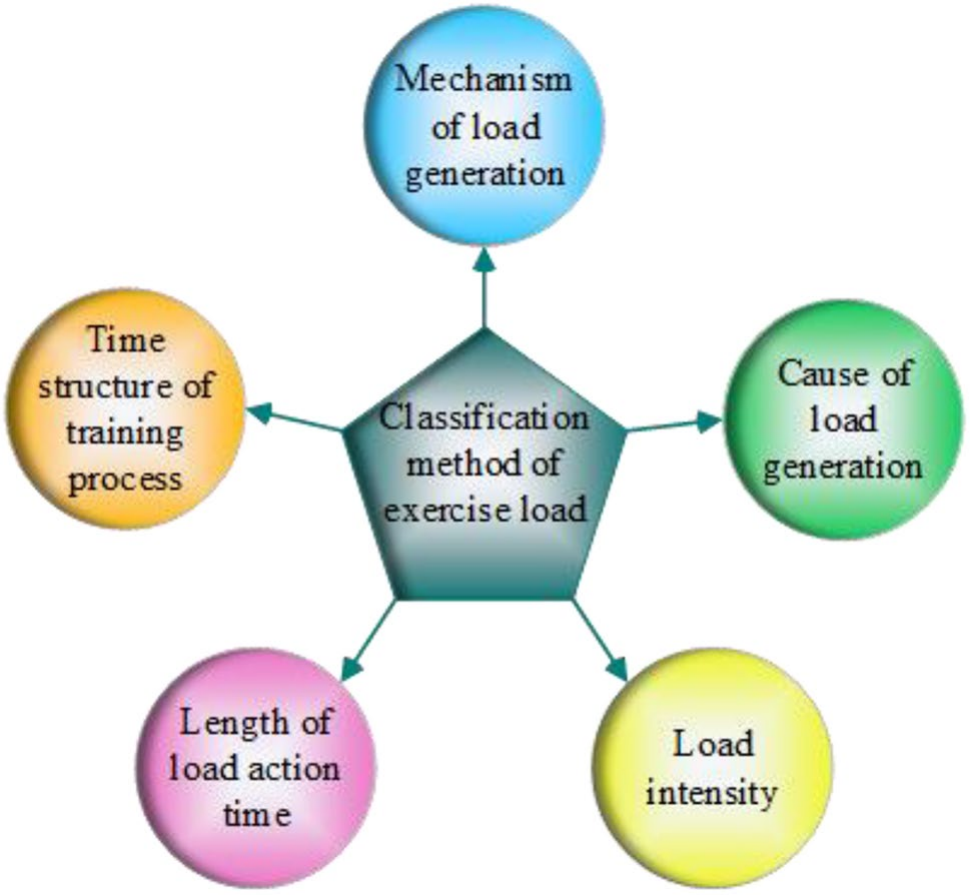
Classification of exercise load.

A particular type of sporting activity created especially for people with lower limb disabilities is sitting volleyball. It follows basically the same regulations as the volleyball competition for athletes. The distinction is that impaired athletes play sitting volleyball while seated on the ground, and their hips cannot leave the ground while competing^10^. The primary arguments used by scholars to question the nature of exercise load are workload, stimulation, and reactivity. Exercise load is defined as a type of workload, stimulation is the form and means of exercise load, and reaction is the physiological or psychological effect produced by exercise load acting on the body, taking into account the connotation and extension of exercise load as well as the needs of training practice^11^. Exercise load, then, is the workload associated with physical activity that stimulates an athlete’s body^12^. Figure 1 illustrates the various classification standards for exercise load.

Five techniques for classifying exercise load are shown in Fig. 1. There are two types of loads that can be generated: internal and external loads, physiological and psychological loads. Training load and competitive load can be distinguished based on the causes of the burden. It can be classified into small load, medium load, large load, and ultimate load depending on how intense the load is. Acute load and chronic load are two categories that can be distinguished based on the length of the load action. The training process can be separated into training classes, days, weeks, months, and years of load, or stage load, cycle load, and year-round load, depending on the time structure^13–15^. When referring to an athlete's body, the term "load intensity" describes the internal response brought on by a specific level of external load stimulation throughout a given period of time or a continuous movement^16^. The advancement of big data technology has made it possible for us to track athletes’ physical condition in real time at this moment. The use of training monitoring during the training process allows coaches to efficiently keep track of players' physical and mental workloads and to predict when they are ready to compete.

The inclusion criteria included membership in the women's sitting volleyball team and signing an informed and voluntary consent to participate in the study. The exclusion criteria were absence from training during the study and withdrawal of consent to participate in the study without giving a reason.

### Analysis of load intensity monitoring index

Neural network (NN) mainly maps the input data to high-dimensional space to better complete “data classification”. Such nonlinear transformation can greatly improve data classification ability. The deeper the NN is, the higher the accuracy is in a certain range. Still, with the continuous increase of network depth, the model training convolutes, resulting in a gradual accuracy decline. Against such a dilemma, ResNet is proposed by adding a linear transformation branch to the traditional NN and replacing the direct fitting basic mapping $H(x)$ with $F\left(x\right)+x$ to fit the residual mapping. The “quick connection” method eliminates the difficulty of deeper NN training^17^. The residual connection mode is described by Eqs. (1) and (2):

1 $$F\left(x\right)=H\left(x\right)-x,$$

2 $$y=F\left(x\right)+x.$$

$F(x)$ represents the residual mapping; $H(x)$ refers to the basic mapping in traditional NNs; $x$ indicates the input data; $y$ means the output of the residual connection. These equations demonstrate how residual connections in ResNet calculate residual mapping $F(x)$ and add it to the input data $x$ to obtain the final output $y$. This approach helps mitigate degradation problems caused by deeper NNs and improves the accuracy of data classification.

Indicators such as heart rate, blood lactic acid (BLA), urea, creatine kinase, and RPE are often used to assess exercise load intensity^18^. Heart rate is the number of heart beats per minute and usually shows resting heart rate (RHR). RHR is the number of heart beats per minute in calm, passive, and quiet states, which is usually used to track the recovery of fatigue or the presence of disease in athletes, and its value is affected by multiple factors such as age, gender, and health status. As the exercise progresses, the RHR should decrease. At the same time, there is a maximum heart rate (MHR). The MHR is the highest heart rate that can still be maintained after reaching the maximum exercise load intensity, at which point oxygen consumption and heart rate cannot increase. During exercise, the intensity of the training load can affect the changes in heart rate. Heart rate is positively correlated with exercise load intensity^19,20^. Intensity can be divided into five stages based on MHR percentage. The first interval stage is the general activity stage, in which the MHR percentage is between 50 and 59%, and the heart rate is less than 119 beats per minute. The heart rate range for the second interval stage is 120 to 139 beats per minute, with an MHR percentage of 60 to 69%, corresponding to low-intensity activities. The heart rate range of the third stage is between 140 and 159 beats per minute, with an MHR percentage ranging from 70 to 79%, which is related to moderate-intensity exercise. The fourth stage corresponds to high-intensity activities, with a heart rate of 160–179 beats per minute and an MHR percentage of 80–89%. The fifth stage, with a heart rate greater than 180 beats per minute and a corresponding MHR percentage exceeding 90%, belongs to extremely intense exercise^21,22^. The variation of exercise load intensity and time has an impact on BLA concentration. By understanding the metabolic changes in lactate production and lactate clearance during exercise, people can understand the characteristics of energy metabolism during exercise and how to control the intensity of exercise^23^. When the training load of athletes is too strong, the breakdown of proteins in muscles will increase, which may lead to hematuria. At the same time, urine output will decrease, renal blood flow will decrease, and blood urea will significantly increase^24,25^. The basic concept of RPE comes from the subjective physical sensation of the human body, that is, the degree of tolerance or subjective sensation to a specific intensity, which is usually described by a 6–20 RPE scale, as exhibited in Table 1^26^.

**Table 1:** RPE subjective sensory exercise load rating table.

Self-sensation	RPE grade	Strength grade	Relative strength [%]
Extremely easy	6	General activities	0.0
	7	Small intensity	7.1
Very easy	8		14.2
	9		21.3
	10		28.4
Quite easy	11	Small and medium strength	35.5
	12		42.6
A little hard	13	Medium strength	50.0
	14		57.1
Hard	15	Medium and large intensity	61.3
	16		71.5
Very hard	17		78.6
	18	Large intensity	85.8
Extremely hard	19		90.0
Spare no effort	20		100

Heart rate monitoring is common in sports training to track players' progress because it is straightforward and simple to get. Blood must be collected for blood-based markers like blood lactic acid. Athletes find it difficult to tolerate repeated blood draws over an extended period of time and will experience some stress as a result. RPE is a crucial tool for tracking and accurately estimating the intensity of exercise load since it sits somewhere between psychology and physiology^27–29^. Due to the unique nature of sitting volleyball, there are more arms and core workouts for the waist and belly than there are for running, jumping, or other whole-body movements. Since heart rate cannot accurately reflect the intensity of a physical training session, the RPE scale must be used to track athletes' physical preparation. Heart rate and RPE are thus chosen in this study as the monitoring indicators of load intensity during the women's sitting volleyball training procedure.

### Experimental test method

The experiment is performed in a training and competition course, and the first week of training and competition lasts for one week. The training week is specially designed by coaches to help athletes develop competitive ability and technical and tactical cooperation. Considering that most of the sitting volleyball players are senior athletes, the morning training session has a low load intensity target and the afternoon training session has a moderate load intensity target, which is very beneficial to the recovery of the players. The main training content of this week includes two-person passing, multi-person passing, multi-person counter-adjustment, reversal, team confrontation, blocking training, serve and receive serve, six-person attack, etc. This is followed by a second week of training, most of which is devoted to technical training sessions. Thursday morning and Sunday are closed. The test is conducted at a disability sports training facility, using a Team heart rate meter (a Polar Team Pro Sensor made in Finland) to track heart rate. Before performing the exercise, the sensor and tablet are placed into the base, each athlete is assigned a sensor, the sensor is fixed to the strap, and the athlete is given the corresponding number. Then the data is synchronized. Finally, athletes are shown how to properly wear Polar watches and assisted in wearing them. The Polar table is removed and the test data after training is exported. To assess each athlete's overall feelings about the sport, the athlete is asked to complete the RPE scale within 10 min of the end of each training session during the second week. To ensure that athletes understand the meaning of each level of the RPE scale, they are given an explanation before using the RPE for the first time. The RPE scale for each athlete is printed out before the training session begins. Ten minutes later, the athletes are shown the scale and their subjective responses are obtained. Ultimately, Excel and Statistical Product and Service Solutions (SPSS) programs are used to input and count the data from the test facility.

To perform statistical analysis and represent statistical significance, the Pearson correlation test is used to conduct statistical analysis on the obtained results, and results with a p-value ≤ 0.05 are considered statistically significant. Specifically, the testing facility's data is inputted, calculated, and analyzed using Excel and SPSS programs. Additionally, the data is statistically tested using the Pearson correlation test to determine if there are significant differences between diverse variables. In the process of the Pearson correlation test, the P-value is an important index used to measure the significance of experimental results. When the P-value is less than the set significance level (0.05), the result is considered remarkable, i.e., the observed difference is unlikely to be caused by random factors. Conversely, if the P-value is greater than the significant level, the result is considered insignificant, i.e. the difference may be caused by random factors.

## Results

### Characteristics of the subjects

**Figure 2:**
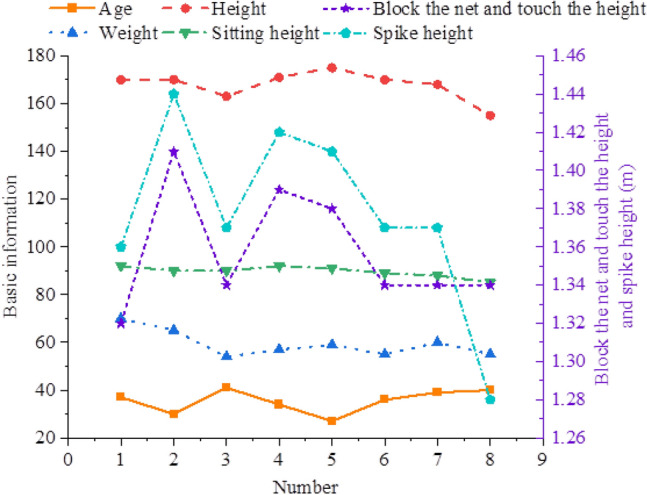
Basic information of athletes.

Eight athletes from a women's sitting volleyball team in a province are the focus of this study. Player No. 5 is the main attacker, followed by Nos. 1, 2, 4, and 6 for assistance attackers, Nos. 3 and 8 for setters, and No. 7 for the receiver. Figure 2 depicts the fundamental situation of these sportsmen.

According to Fig. 2, these athletes are 36 and a half years old on average, with the oldest being 41 and the youngest being 27. The highest and lowest heights are 175 cm and 155 cm, respectively, with the average height being 168 ± 6 cm. The average sitting height is 90 ± 2 cm, and the average weight is 59 ± 6 kg. The maximum blocking height is 1.41 m, the lowest is 1.34 m, and the average blocking height is 1.36 ± 0.03 m. The maximum spike height is 1.44 m, the lowest is 1.28 m, and the average spike height is 1.38 ± 0.05 m. Table 2 outlines the statistical results of the subjects' characteristics in this study.

**Table 2:** Characteristic statistics of the subjects.

Indicators	Average	Maximum value	Minimum value	Standard deviation
Age (years)	36.5	41	27	6.75
Height (cm)	168	175	155	6.53
Seating height (cm)	90	62	111	2.23
Body weight (kg)	59	51	70	6.25
Blocking height (m)	1.36	1.41	1.34	0.03
Spike height (m)	1.38	1.44	1.28	0.05

### Performance evaluation

**Figure 3:**
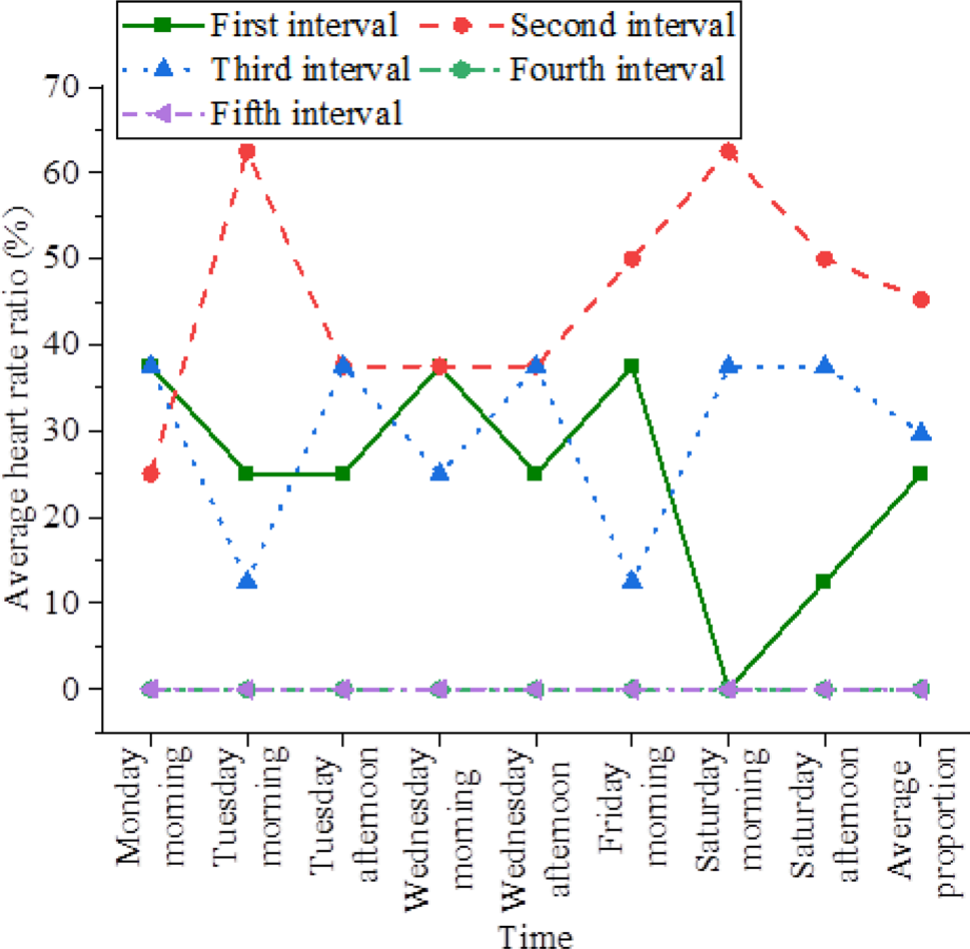
The distribution of athletes' average heart rate during the training competition week.

The average heart rate distribution of athletes during the training competition week is shown in Fig. 3.

In Fig. 3, the first, second, third, and fourth periods are the training period of the women's volleyball team, which is also the experimental period designed in this study, namely the experimental group results. The fifth interval is the control group results, namely the statistical results of heart rate when the women's volleyball team is not trained. The above data results show that the first, second, and third periods of the training competition week are the most evenly distributed periods for players' average heart rate. The distribution of the average heart rate in the second period is the highest (45.3%), and low-intensity exercise is a matching training type. Next is the third period, accounting for 29.7% of the total, with moderate-intensity exercise being the corresponding training intensity. It can be concluded that during the competitive training week, the exercise load intensity of the women’s volleyball team is mainly low intensity and a small amount is moderate intensity.

**Figure 4:**
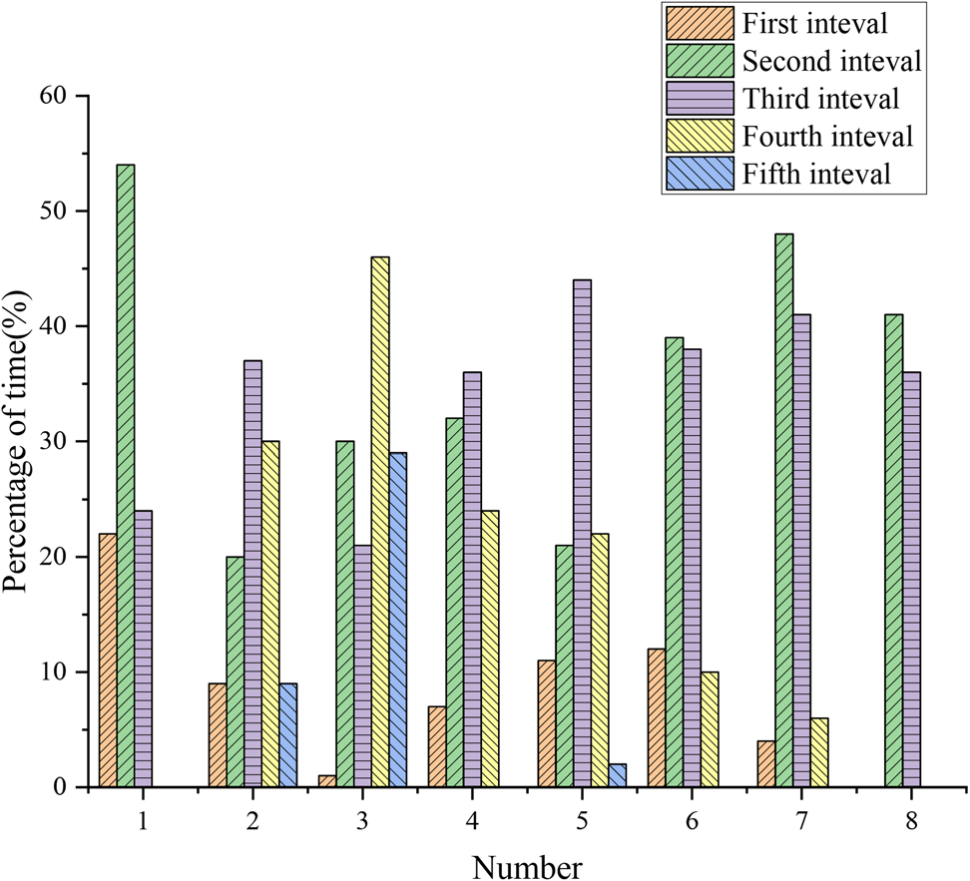
Time distribution of athletes’ heart rate interval on teaching competition day.

The statistics of the time distribution of athletes' heart rate intervals on the training and teaching competition day are shown in Fig. 4.

Figure 4 demonstrates that player No. 8 has the highest heart rate in the first interval on the last day of the week, accounting for 23% of the total time, and its corresponding position is the second level, indicating that her resting time is longer. This is because her height limits the number of nets she can use and she moves less, resulting in a lower exercise intensity. The most players in the third and fourth intervals are No. 2, No. 3, and No. 5, corresponding to the secondary attack, the main attack, and the receiving position respectively. They actively participated in the game, and Nos. 2 and 5 continued to smash the ball. The third interval also has 37%, 22%, and 44% of athletes in positions 2, 3, and 5, while the fourth interval has 30%, 47%, and 22% of athletes in positions 2, 3, and 5, respectively. Their heart rates account for 9%, 29%, and 1% respectively, are in the fifth interval, possibly due to the conditions during the competition. On the last day of the week, the amount of exercise is generally low, but the load intensity of the No. 2 secondary attacker, No.3 receiver, and No.5 main attacker is higher than that of the other players.

**Figure 5:**
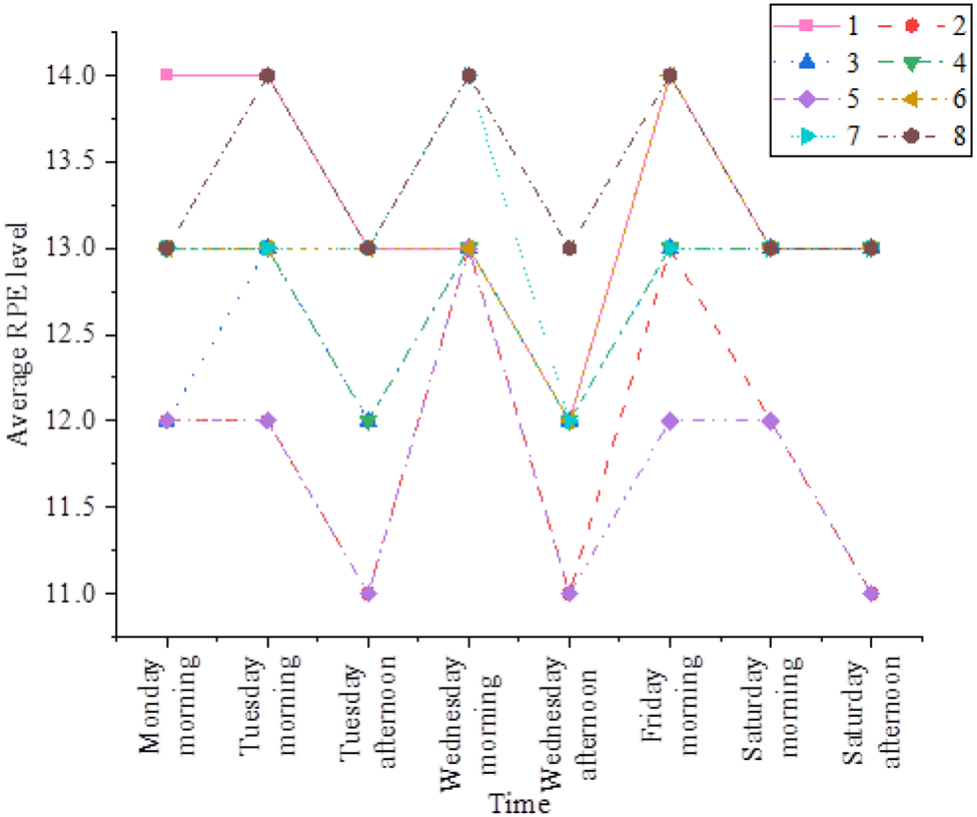
RPE results of athletes in training competition week.

The RPE results of athletes in the first week of training competition week are shown in Fig. 5.

According to Fig. 5, during the training competition week, athletes' RPE levels essentially fluctuate in waves. This is because the coach divides the training week into two parts and schedules lighter training on Wednesday or Thursday to allow for adjustments and the athletes' recovery from high-intensity training. When observing the women's sitting volleyball team's physical training, it is discovered that while the coaches utilize the same training material, weight, amount of exercises, and groups for new and experienced players, the RPE results are not the same. The training program can be suitably altered based on the athletes' feedback on training intensity, and a more specialized training program can be created for the athletes' age, speciality, and difficulty.

**Figure 6:**
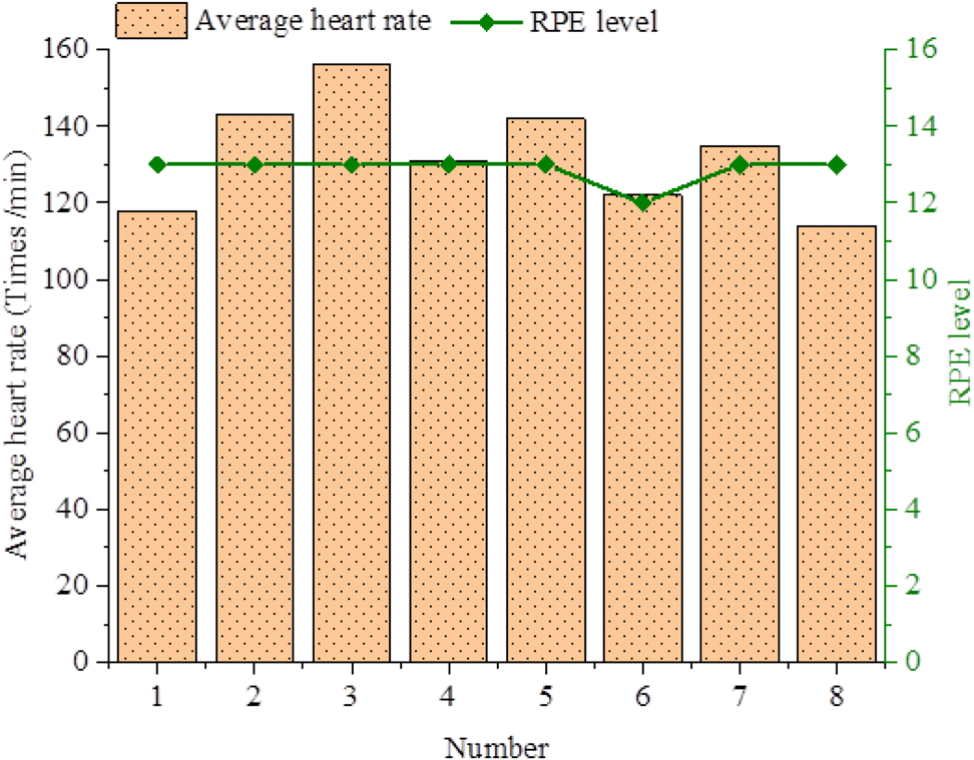
Average heart rate and corresponding RPE level in a technical training class on Wednesday afternoon.

Take Wednesday afternoon of the second week of technical training class as an example, the average heart rate of athletes and the corresponding RPE level statistics are shown in Fig. 6.

**Figure 7:**
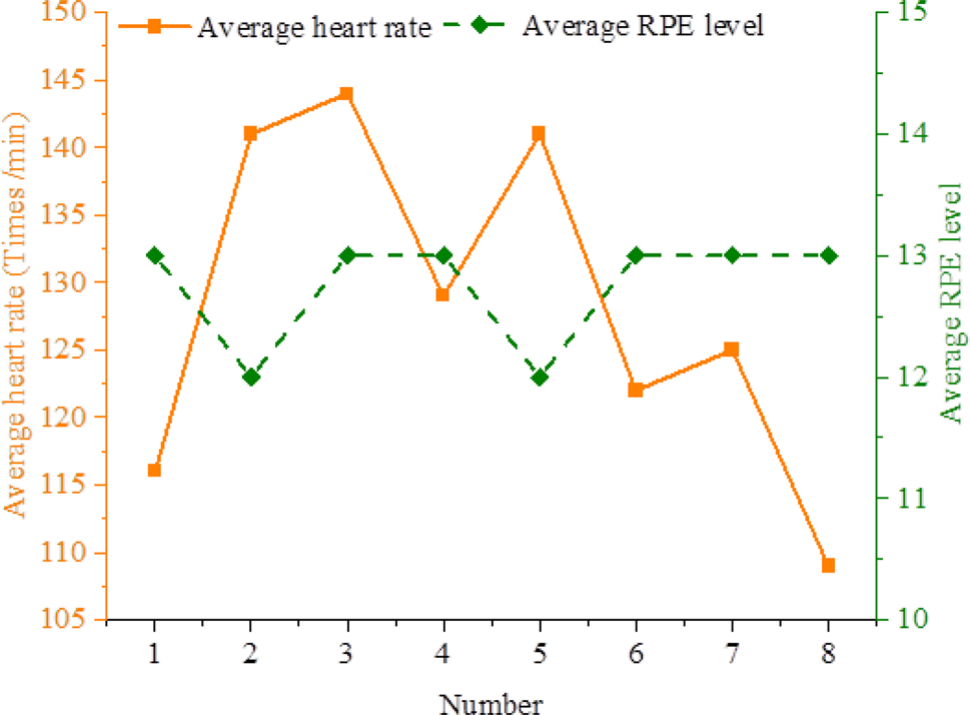
Results of overall average heart rate and average RPE level in technical training class.

According to Fig. 6, the No. 3 athlete's average heart rate in the technical training class on Wednesday afternoon is 156 beats per minute, and the No. 8 athlete's average heart rate is 114 beats per minute. Only the No. 6 athlete has an RPE level of 12, which is associated with reasonably simple medium- and small-intensity training. The majority of RPE levels are 13, which is associated with a little tough moderate-intensity exercise. Figure 7 displays data from the second week of technical training class on the average heart rate and RPE levels.

According to Fig. 7, the average heart rates of athletes No. 1, No. 4, No. 6, No. 7, and No. 8 are 116, 129, 122, 125, and 109 beats per minute, respectively. This indicates that their training falls under the category of low-intensity exercise, yet subjectively, the RPE level is 13. The RPE level is correlated with the average heart rates of athletes No. 2, No. 3, and No. 5. The two youngest athletes, Nos. 2 and 5, also have the lowest RPE levels of the three. The average heart rate of the third athlete, an older athlete, matches the RPE level, indicating that the third athlete is in good health.

The correlation analysis between athletes' average heart rate and RPE is shown in Table 3.

**Table 3:** Correlation analysis between athletes' average heart rate and RPE.

Athlete number	Pearson correlation (R-value)	P-value
No. 1	0.771	0.014
No.2	0.955	0.009
No. 3	0.855	0.008
No. 4	0.91	0.007
No 5	0.856	0.0095
No. 6	0.727	0.015
No. 7	0.713	0.017
No. 8	0.92	0.0087

Table 3 demonstrates that there is a substantial correlation between the average heart rate and RPE of the three athletes, with the correlation coefficients of athletes No. 1, No. 6, and No. 7 being 0.771, 0.727, and 0.713, respectively, with their P-values all falling within the range of 0.01P0.05. The remaining five athletes' p-values are all less than 0.01, demonstrating that there is a highly significant link between their average heart rates and RPE. RPE can be utilized as a monitoring gauge of the intensity of the training load for women's sitting volleyball players because, generally speaking, the average heart rate of these athletes is positively connected with RPE.

## Discussion

Based on big data technology, this study designed a load intensity detection platform for women's sitting volleyball training and carried out corresponding evaluation experiments with the platform. This study found a positive correlation between RPE and the average heart rate of women's sitting volleyball players. In contrast to other similar studies, Costa et al.^30^ measured the internal training load of six elite female soccer players over 90 weeks to examine the relationship between RPE and training stimulation. They found a strong association between the two and a significant difference between each RPE value and the percentage associated with peak heart rate^30^. This finding was consistent with the findings of this study and further validated the relationship between heart rate and RPE. Ouergui et al.^31^ studied the effects of the competition area and type of judo training on female judo athletes' physiological response and RPE. They found that although the type of training or area size did not have an appreciable effect on lactic acid acidity before each race, heart rate before each race, average heart rate per race, and RPE score, it may alter the physiological response of female judo athletes during competition^31^. This study demonstrated that RPE was insensitive to race trends, even though it did not support the hypothesis of a correlation between heart rate and RPE. Rago et al.^17^ used an 18Hz accelerometer to track the heart rate and RPE of men's ice hockey players to investigate the effects of RPE-based load monitoring in elite ice hockey training. They found a small association between RPE and exercise intensity measures^17^. Contrary to the findings of this study, their study showed a small association between RPE and training intensity. The study was based on ice hockey players, and there were obvious differences in sitting volleyball players' training and playing patterns.

There are some limitations to this study. First, the sample size is relatively small and may not be representative of the characteristics of the entire population of women's sitting volleyball players. Second, this study only focuses on the relationship between RPE and average heart rate, and can also consider the influence of other physiological parameters, training content, and technical factors on RPE. Furthermore, in future studies, other applications of using load intensity monitoring platforms in sports can be explored to gain a deeper understanding of the relationship between athletes' training load and performance, positively improving training effectiveness and preventing sports injuries.

## Conclusions

This study explores the concept and definition of "exercise load intensity", discusses the classification and monitoring indicators of "exercise load", and selects heart rate and RPE as monitoring indicators of women's sitting volleyball training research. With the support of big data technology, a platform was designed to monitor the exercise load intensity of women's sitting volleyball. The results show that, firstly, the RPE level of athletes fluctuates during the training competition week, which means that they have different perceptions and adaptability to the training intensity. This finding provides an important basis for coaches to make training plans. According to the feedback and feelings of different athletes, the intensity of training is adjusted to ensure that their bodies adapt and recover. Secondly, although coaches use the same training materials, weights, reps, and sets, RPE results vary among athletes. This suggests that individual differences in athletes play a vital role in training. Therefore, athletes' age, professionalism, and difficulty should be fully considered in the formulation of training programs, and personalized training programs should be designed to better meet their training needs and goals. Finally, this study reveals that the average heart rate of women's sitting volleyball players is positively correlated with RPE, which indicates that heart rate can be used as an effective indicator to monitor the level of training load. Monitoring the athletes' heart rate changes makes it possible to understand their physical responses and load levels during training. This helps coaches and athletes grasp the training effect, adjust the training plan in time, and ensure scientific and effective training.

## Data Availability

The datasets generated during and/or analysed during the current study are available from the corresponding author on reasonable request.
